# Participants’ accrual and delivery of HIV prevention interventions among men who have sex with men in sub-Saharan Africa: a systematic review

**DOI:** 10.1186/s12889-018-5303-2

**Published:** 2018-03-20

**Authors:** Daniel Nyato, Evodius Kuringe, Mary Drake, Caterina Casalini, Soori Nnko, Amani Shao, Albert Komba, Stefan D. Baral, Mwita Wambura, John Changalucha

**Affiliations:** 1 Sauti Program, National Institute for Medical Research, P.O Box 1462, Mwanza, Tanzania; 2 Sauti Program, Jhpiego Tanzania - an affiliate of Johns Hopkins University, P.O Box 9170, Dar es Salaam, Tanzania; 3 Key Populations Program, Center for Public Health and Human Rights, Department of Epidemiology, Johns Hopkins Bloomberg School of Public Health, E7146, 615 N. Wolfe Street, Baltimore, MD 21205 USA

**Keywords:** HIV intervention, Accrual, Delivery, Men who have sex with men, sub-Saharan Africa

## Abstract

**Background:**

Across sub-Saharan Africa (SSA), HIV disproportionately affects men-who-have-sex-with-men (MSM) compared with other men of the same age group in the general population. Access to HIV services remains low among this group although several effective interventions have been documented. It is therefore important to identify what has worked well to increase the reach of HIV services among MSM.

**Methods:**

We searched MEDLINE, POPLINE and the Web of Science databases to collect published articles reporting HIV interventions among MSM across sub-Saharan Africa. *Covidence* was used to review the articles. The review protocol was registered in International Prospective Register of Systematic Reviews (PROSPERO) - CRD42017060808.

**Results:**

The search identified 2627 citations, and following removal of duplicates and inclusion and exclusion criteria, only 15 papers were eligible for inclusion in the review. The articles reported various accrual strategies, namely: respondent driven sampling, known peers identified through hotspot or baseline surveys, engagement with existing community-based organizations, and through peer educators contacting MSM in virtual sites. Some programs, however, combined some of these accrual strategies. Peer-led outreach services were indicated to reach and deliver services to more MSM. A combination of peer outreach and mobile clinics increased uptake of health information and services. Health facilities, especially MSM-friendly facilities attract access and use of services by MSM and retention into care.

**Conclusions:**

There are various strategies for accrual and delivering services to MSM across SSA. However, each of these strategies have specific strengths and weaknesses necessitating combinations of interventions and integration of the specific context to inform implementation. If the best of intervention content and implementation are used to inform these services, sufficient coverage and impact of HIV prevention and treatment programs for MSM across SSA can be optimized.

**Electronic supplementary material:**

The online version of this article (10.1186/s12889-018-5303-2) contains supplementary material, which is available to authorized users.

## Background

Men who have sex with men (MSM) face a disproportionate burden of HIV infection globally [1–3]. HIV prevalence reported among MSM worldwide remains high [3], in contrast to the declining trends reported among men in similar age group [4]. The high burden of HIV among MSM is also evident across sub-Saharan Africa with generalized HIV epidemics. For example, the 2012 Global AIDS Response Progress Reporting (GARPR) data show that the highest median HIV prevalence among MSM worldwide came from Western and Central Africa (19%), and East and Southern Africa (15%) [5]. As the world aims at ‘ending the HIV epidemic by 2030’ [6], the high HIV prevalence among MSM is a cause for concern. Indeed, the UNAIDS has cautioned that such a goal cannot be reached without overcoming issues that undermine effective HIV response for key populations, including MSM [7].

Uptake of HIV prevention services is critical to achieving a decreased HIV incidence and eventually prevalence among MSM [4]. There are various HIV interventions, including pre-exposure prophylaxis, universal access to antiretroviral treatment, HIV testing, and condom use that are recommended by the World Health Organization (WHO) and are implemented globally [8, 9]. Yet, uptake of these interventions has not successfully reduced new HIV infections among MSM. In sub-Saharan Africa, where cultural values stigmatize and even criminalize same-sex practices, reaching MSM and delivering HIV interventions is challenging [10, 11]. As a result, coverage of HIV prevention services for MSM is often suboptimal, and MSM continue to experience suboptimal health outcomes due to limited-access to evidence-based HIV preventive services.

There are well-documented barriers to uptake of HIV interventions in sub-Saharan Africa. At a structural-level, laws that sanction same-sex practices lead to social prejudice, threats and violence against people thought to be homosexual. This deters them from seeking health care and treatment services [12–14], as well as using healthcare services seen to be friendly to MSM as they hide their sexual orientation [15]. Studies report inconveniences (i.e. unfriendly location of health service), breach of confidentiality and privacy, fear of stigma and discrimination, as well as long waiting time [10, 12, 14, 16], to keep MSM from accessing available health services. At individual-level, fear, perceived risk, embarrassment in discussing with health providers on their sexual orientation, limit the possibility of seeking services and receiving proper care. In response, researchers and practitioners have tried several strategies to reach this population and devised strategies to deliver the services. However, these approaches are not uniform in implementation contexts, aims, and outcomes.

This review synthesizes evidence on accrual strategies for MSM (i.e. how to reach and engage them in health care) and service delivery strategies (i.e. how are the HIV information and services provided) for HIV prevention. The primary aim was to systematically review and describe accrual, delivery strategies and uptake of services among MSM in sub-Saharan Africa where homosexuality is both socially and politically unacceptable.

## Methods

We registered the protocol for this systematic review in PROSPERO and received a registration number CRD42017060808.

### Search strategy

This review set to identify studies reporting HIV prevention interventions among MSM published in English from March 15th, 2007 to April 14th, 2017. We searched MEDLINE, POPLINE and the Web of Science databases to collect published articles. A combination of topics (HIV or HIV/AIDS), population (“men who have sex with men”, “bisexual men”, “homosexual men”, “gay”, “MSM”, “male sex workers”) and a context (sub-Saharan Africa) were used to perform a search of relevant articles. This strategy helped to get and synthesize a wide range of research evidence than focusing on a single intervention. A combined search strategy in MEDLINE is provided as a supplementary material (Additional file 1).

### Inclusion and exclusion criteria

The review included: primary studies reporting HIV interventions among MSM, focused on MSM, reported at least one review result, published in a peer-reviewed journal, and was in English. In addition, with the establishment of the first WHO’s “consolidated guidelines on HIV prevention, diagnosis, treatment, and care for key populations” in 2014 [9], this review aimed at gathering studies conducted within ten years (March 15th, 2007 – April 14th, 2017). Excluded studies were those: conducted outside sub-Saharan Africa or were a continuing intervention.

### Study selection

Two independent reviewers (DN, EK) screened the titles and abstracts of all articles. The two reviewers used *Covidence* (https://www.covidence.org), an online-tool developed for systematic reviews by the Cochrane Collaboration to screen and assess the titles and abstracts based on a guidance tool for assessing eligibility. We assessed the full text for both eligible studies as well as those that the two reviewers could not decide based on titles and abstract alone. In addition, if at least one reviewer considered the article as relevant, we assessed its full text. A third reviewer (MW) joined the discussion and decision reached.

### Quality

Two review authors (DN, EK) adapted and used a STROBE combined checklist [17] for quality assessment of cohort, case-control and cross-sectional studies to examine included studies. The two reviewers assessed all included studies for possible risk of bias on a scale of high, low and unclear. Items of the assessment included: applicability or appropriateness of the sample, the applicability of the results, completeness of reporting the design and methods, completeness of result reporting, declaration of funding, and disclaimer of interest among researchers. We did not exclude any study following the quality assessment. Reviewers’ differences regarding the quality of included articles were resolved through a discussion without the third reviewer.

### Data extraction and synthesis

DN extracted the data and recorded into a pre-developed form. DN and EK piloted this form before the search. EK corroborated the accuracy and precision of extraction by checking all the data against studies. Table 1 shows a summary of extracted results with names of the author, year published, and country of study, study aims, study design, accrual strategy, delivery strategy and or results.

**Table 1:** Summary of studies reporting accrual and delivery strategies of HIV interventions among MSM in SSA

Author/Study country	Study Aims	Study Design	Study population characteristics	Participant Accrual	Delivery strategy	Uptake/Results
(Adebajo et al., 2015) [30] Nigeria	To evaluate the effect of three strategies in increasing uptake of HIV counseling and testing (HCT) among male most-at-risk population	Cross-sectional	Media age Arm I: 28 years, Inter quartile range (IQR) 22–36 years Arm II: 30 years, IQR 24–40 years Arm III: 28 years, IQR 24–34 years	Not explicit	Arm1: Key opinion leaders (KOLs) referring MSM to health facilities for HTC -facility based Arm2: KOL referring MSM to nearby HTC team - community based Arm 3: mobile M-MARPs peers conducting the HTC -community based	Arm1: 1988 MSM reached Arm2: 14,726 MSM reached Arm3: 14,895 MSM reached Proportion of new HIV diagnosis were: Arm1 = 8%, Arm2 = 3%, and Arm3 = 13%
(Baral et al., 2015) [31] Malawi	To evaluate the utility of respondent-driven sampling as an implementation tool for engaging MSM in HIV intervention	Prospective cohort	Participants with tertiary education decreased from 28% in the first 10 waves to 9% in wave 26 (*P* < 0.001) Mean age also decreased among participants from earlier waves to that of later waves (P < 0.001)‡	Respondent-driven sampling (RDS)	HIV prevention and care services were provided at a dedicated facility established by the community-based organization- facility based	MSM were more likely to report having tested for HIV in waves 0–4 (82.9%) than in waves 20–26 (47.7%) 80% of MSM correctly reported their HIV status in earlier waves, while only 25% correctly reported their status in later waves.
(Batist, Brown, Scheibe, Baral, & Bekker, 2013) [21] South Africa	To reach MSM in five Cape Town townships, disseminate HIV-prevention information and supplies, and promote the use of condoms and HIV services.	Prospective cohort (Pilot)	Median age 24.5 years, IQR of 21–29 years 82.3% of participants were gay identified 10.4% self-identified as bisexual	Use of known peers to bring friends or Known peers identifying friends through hotspots or baseline survey contacts	The intervention activities like training, debates and condom and lubricant provision were conducted in safe spaces - community based Where required, the MSM were referred to MSM-friendly facilities for further care-facility based	Participants reported increased access to HIV prevention services i.e., condom and lubricant use. Reported reduced feelings of loneliness and social isolation
(CHARURAT et al., 2015) [28] Nigeria	To examine acceptability of a treatment as prevention (TasP) strategy among HIV-infected MSM using a Trusted Community Centre providing comprehensive HIV prevention and treatment services to MSM in Abuja, Nigeria.	Prospective cohort	52.4% above 25 years 82.1% had at least senior secondary or higher education 69.0% self-identified as bisexual 31% self-identified as gay or homosexual 68.8% not on antiretroviral therapy (ART)	Respondent-driven sampling (RDS)	HIV related services were provided at a dedicated facility (trusted community center) -facility based	Of 186 HIV positive individuals, 128 (68.4%) were not on ART and were offered TasP. Individuals who were not on ART at the time of enrolment were more likely to have not disclosed their sexual identity to health care providers (70.1% vs. 45.6%, P, 0.01) and to have not discussed HIV with their closest friends (81.2% vs.62.5%, *P* = 0.01).
(Geibel, King’ola, Temmerman, & Luchters, 2012) [32] Kenya	Evaluate the impact of a peer-driven HIV intervention on male sex workers who sell sex to men in Mombasa, Kenya.	Prospective cohort	Baseline Median age 26 years, IQR 22–31 years 58.3% self-identified as bisexual Follow-up Median age 23 years, IQR 21–27 years 56.1% self-identified as bisexual	Pool of MSM who had participated in a previous baseline study	The HIV prevention and care related services were offered at a drop-in-center (DIC) (dedicated facility) established by International Centre for Reproductive Health (ICRH) Kenya. This DIC also acted as a safe space- facility based	Increased consistent condom use with both paying clients (35.9%e50.2%, *p* < 0.001) and non-paying male partners (27.4%e39.5%, p¼0.008). Peer educator contact was also associated with improved HIV knowledge and use of water-based lubricants.
(Graham et al., 2015) [23] Kenya	To promote care engagement and antiretroviral therapy (ART) adherencefor MSM in coastal Kenya	Prospective cohort (Pilot)	All participants were aged between 24 and 42 years Education years between 4 and 14 years	Local CBOs, health providers, and informal peer outreach	Research-based health facility provided HIV related prevention and care services facility based	Of 10 ART-naïve participants who enrolled in the pilot, eight completed follow-up with no adverse events reported.
(Möller et al., 2015) [20] Kenya	To describe changes in sexual risk behavior among Kenyan MSM who receivedregular risk reduction counseling (RRC).	Prospective cohort study - using a HIV-1 negative and HIV-1 positive MSM	Median age 25.2 years, IQR 21.5–29.7 years 53.8% had primary or no formal education	Use of known peers to bring individuals via personal networks and from known hotspots	Not clear from the article.	Participants (HIV-1 negative & HIV-1 positive) reported decreased number of sexual partners and unprotected anal intercourse
(Mulongo et al., 2015) [29] DR Congo	To reduce the risk and impact of HIV in the DRC through community- and facility-based prevention, counseling and testing,and treatment strategies aimed at high-risk populations by increasing access and utilization of HIV intervention and care services	Case study	Not specified in the paper	Venue-based recruitment sessions Virtual sites and text messaging platforms	HIV related services were offered through mobile venue-based outreach service- Community based The referrals were done to a key population-friendly health facility and other local facilities for further care- facility based	Was able to reach 2621 MSM with targeted prevention messaging in 2013, and provided testing and counseling to 4366 MSM from October 2012 to June 2014.
(Singh et al., 2012) [24] Kenya	Assess acceptability of venue-based approach for providing VCT	Cross-sectional	78.6% had at least 25 years 75.6% had primary or no formal education	Use of known peers (community informants) to recruit MSM from known hotspots	Services were offered in mobile outreach clinics at or near the venues -community based Facility based services were offered to those who tested HIV positive - facility based	HIV prevalence was higher in this study compared to individual’s sampled in the 2008–2009 KDHS, suggesting the appropriateness of venue-based sampling in reaching stigmatized populations
(Wirtz et al., 2015) [27] Malawi	Testing the feasibility of providing a combination HIV preventionintervention (CHPI) for MSM in Malawi.	Prospective study, Before and after evaluation	57.3% aged between 18 and 25 years 65.0% had completed secondary or higher education 68.9% self-identified as gay or homosexual	Respondent-driven sampling and through MSM identified during a previous baseline study	Services were provided at a dedicated facility created by a community-based organization (CBO)- facility based Referrals were done to MSM-friendly local hospitals and to Johns Hopkins antiretroviral therapy and sexually transmitted infection clinic.- facility based The MSM-friendly facilities had received sensitization training -facility based	Improved reported condom use at last sex (from 62.5% at baseline to 77.0% at follow-up 3). Increased disclosure of sexual orientation from 25% in follow-up 1 to 55% in follow-up 3.
(Williams, Carney, Plüddemann, & Parry, 2014) [26] South Africa	To provide a descriptive summary of programmatic work targeting substance-related HIV riskbehavior among MSM in South Africa.	Cross-sectional	78.6% aged at least 25 years 75.6% had completed secondary or higher education	Engagement with a local NGO and Peer outreach	Community-based outreach - community based	3475 drug-using MSM were reached through community outreach. 745 MSM tested for HIV and received results. 239 MSM were referred from HTC to other services
(Williams, Carney, & Parry, 2016) [25] South Africa	To test whether an intervention aimed at MSM who use substances (alcohol and other drugs) could affect risky substance use and sexual behavior.	Cross-sectional	Median age 27 years, IQR 18–49 years Median number of years of education 12, IQR 7–13 years	Engagement with a local NGO and Peer outreach	Mobile community-based outreach and provision of information on HIV/AIDS, substance use, and safer sex practices-community based	Contributed to reduction in in the proportion who used cannabis and ecstasy including use of drugs during sex (knowledge about risk reduction strategies increased)
(Dramé, Crawford, Diouf, Beyrer, & Baral, 2013) [22] Senegal	To assess the feasibility of implementing a community-driven HIV prevention study in Senegal.	Prospective cohort	Mean age 28 years and 50% of participants ranged between 23 and 32 years 47.9% had secondary or university education	Through engagement with a CBO and through peers to accrue and retain MSM in Senegal for 15 months	Used mobile clinics to provided syndromic diagnosis and treatment of STI at the site (or on-site) community based Referral for treatment and follow-up were done to local health facilities- facility based	HIV prevalence at baseline was 36.0% (43/114), with cumulative HIV prevalence at study end being 47.2% (51/108).
(Green, Girault, Wambugu, Clement, & Adams, 2014) [11] Ghana	To assess the level of coverage of HIV prevention program using traditional peer-based approaches versus social media outreach.	Case study	Mean age of peer recruiters 25.5, standard deviation 6.9 years 55.4% of participants were age group 18–25 years	Virtual sites based approaches and Venue-based contacts	Web-based	15,440 MSM reached through social media approaches. 12,804 MSM reached through peer-based outreach. The total reach was about 92% of MSM in Ghana.
(Girault et al., 2015) [33] Ghana	To assess the feasibility of using a social network strategy in complementing a peer outreach approach in referring high-risk MSM to HTC services	Cross-sectional study	Not specified in the paper	Respondent-driven sampling (RDS)	Local government owned health facilities in the study setting - facility based	166 MSM reached and referred to HTC in 3 months. 62.7% reported no recent exposure to peer educators. 61.5% unaware of recent HIV status.

We used a narrative synthesis to analyze each retrieved paper. A narrative synthesis is an approach to the systematic review and synthesis of findings from multiple sources and relies primarily on the use of text to summarize and explain the findings [18]. This synthesis is used when statistical meta-analysis or meta-ethnography for qualitative studies is not possible due to extreme heterogeneity in the methodological descriptions of available studies [18, 19]. A narrative synthesis was appropriate for this systematic review given an initial scoping that revealed that the literature was too heterogeneous to allow other forms of synthesis.

## Results

**Fig. 1:**
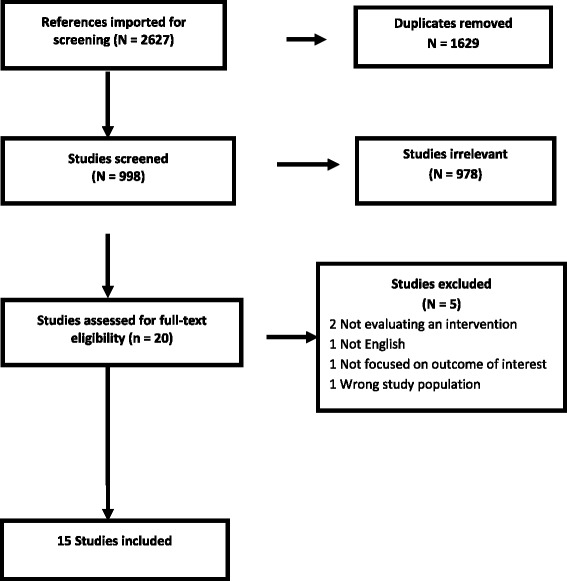
A Prisma flow diagram for article selection

In total, 2627 papers were obtained by searching the online publication databases. After removing duplicates, the search revealed 998 articles. Of these, 978 were excluded based on a review of titles and abstracts, as they were not relevant to our research question. Twenty (20) articles were retrieved for full-text assessment. Of these, five records were excluded because; two were not evaluating an intervention, one did not focus on the results of interest, one did not focus on MSM, and one was not in English. In total, 15 articles were included in this review. Figure 1 below presents a PRISMA flow diagram for selection of articles.

### Characteristics of included studies

Table 2 characterizes the 15 studies included in this review, which represents four regions of SSA. Six studies (40%) were from West African countries, four (26.7%) from Southern Africa countries, four (4) from East Africa (26.7%), and one (6.6%) from Central Africa. Included studies came from seven countries, with Kenya (four studies), Nigeria, and South Africa (three studies each) having the most representation of published articles with a focus on accrual and delivery of interventions. Of note, there were no eligible studies published between 2007 and 2011, and most (60%) were from 2015 or after.

**Table 2:** Characteristics of included studies (*N* = 15)

Article characteristics	*N* (%)
Study country	
Kenya	4 (26.6)
South Africa	3 (20)
Nigeria	3 (20)
Ghana	2 (13.3)
DR Congo	1 (6.7)
Malawi	1 (6.7)
Senegal	1 (6.7)
Publication year	
2012 - 2017	15 (100)
2007 - 2011	0 (0)

### Assessment of risk of bias in included studies

The assessment showed that over half of the studies (*n* = 9/15) had a high-risk of bias in terms sampling [20–28]. These studies had reached their participants through local Non-Governmental Organizations (NGOs), time-venue sampling or had included convenient samples. Except for one study which was unclear [29], 5 articles were assessed as having a low risk as they used multiple sources for accruing participants or used respondent-driven sampling with multiple waves [11, 30–33]. On the completeness of reporting, we found that one study [30] did not report how study participants were obtained and engaged in the intervention. All studies reported sources of funding and declared potential conflicts of interest.

### Participant accrual strategies

Methods of participant accrual are key to the nature and extent of intervention reach. This review has identified five different strategies used to reach and accrue participants:

Two studies used ***respondent-driven sampling*** in getting their participants [28, 34]. As an entry point, implementers approached known MSM in the community (community-based convenience sampling) and explained the purpose of the contact. Those willing to take part were considered as “seeds”. Every seed taking part in the study received coupons with which they had to recruit up to 3 eligible peers. The recruited peers also recruit others. This recruitment continued until when peers sent out to recruit others could not yield anymore [33].

Two studies used a ***venue-based approach*** to recruit participants [24, 29]. The recruitment began with mobilizing peer educators (i.e., MSM recruited and trained to offer specific information and services to their peers) from known MSM hot spots. In a study conducted in DR Congo [29], program staff shared project aims with contacted MSM at the hot spot and their responsibilities as peer recruiters if they accepted to join the project. The program trained recruiters to provide the information of interest to their peers. For example, peer recruiters received training for 5 days on HIV, family planning strategies to protect female partners of MSM and sexual health [29]. A study in Kenya [24] identified settings and events that MSM are believed to frequent and meet new sexual partners. At those places, researchers interviewed key informants (that is, owner, manager, or someone familiar) to get information about the place and about people who socialize at the place, including MSM.

In settings with baseline data, a ***pool of MSM who had taken part in baseline studies*** served as a sampling frame to recruit prospective study participants or reach program beneficiaries [20, 21, 27, 32]. For example, a pilot community-based HIV prevention program among MSM in 5 townships in South Africa, identified and recruited one MSM community leader from previous research in each study site [21]. This MSM community leader helped to identify peers through peer outreach and venue-based contacts. In Blantyre, Malawi, a program that aimed to test the feasibility of combination HIV prevention among adult MSM, invited participants from the baseline study to take part in the 12-month prospective study.

***Engagement with community-based organizations*** (CBOs) that work with MSM locally is a strategy used in three studies included in this review [22, 25, 26]. This strategy benefits from rapport built between NGOs and the beneficiaries to recruit the MSM for other HIV interventions. The studies used outreach workers from local MSM NGO to recruit participants.

Three studies accrued participants through ***virtual sites and text messaging platforms*** [11, 29, 33]. While Mulongo and colleagues used this strategy besides venue-based strategy [29], studies by Girauti [33] and Green [11] had solely used virtual sites to reach and mobilize MSM. These studies used different strategies to start the recruitment. Girauti and colleagues [33] approached counselors and asked them to identify and select a first batch of “seeds” from new or their former clients who previously accessed HIV prevention services. Each seed recruited three MSM (friends) from his social media network and referred them to the setting where they received HIV preventive and treatment services. It was important that recruiter knew the person he recruited by name or nickname, and who was easy to contact. If the seed did not provide any referral within 14 days, the seed became dormant, and new seed recruited. Green and colleagues [11] started recruitment by approaching local CBOs for recommendations of MSM leaders who might be at the center of MSM networks. Three MSM in different towns hired as community liaison officers (CLOs), trained on the use of mobile phones and laptop computers for five days. The CLOs created new profiles on virtual sites visited by MSM and provided information and linked them to available preventive and support services. Mulongo and colleagues [29] used peer educators who worked in the project. The peers sent text messages using coded language, known within MSM communities. The social media used were Facebook, E-mail and short message service (SMS), in which they communicated with their peers about testing events.

A pilot intervention to promote care engagement and adherence among HIV-positive MSM [23] used a ***combination of local MSM organizations, health service providers, and informal peer outreach*** to identify participants.

### Delivery and uptake of interventions

This analysis shows that HIV service delivery for MSM in the studies can be categorized into three delivery strategies, namely: virtual sites, community-based, and facility-based service delivery. Some studies have combined these strategies to increase the numbers of people receiving the services.

### Virtual-site service delivery strategy

Virtual site service delivery was defined as the provision of health-related services including behavioral change communication and information to individuals or groups of people through virtual sites or internet based applications. Our search identified two studies that used ***virtual site delivery*** strategy [11, 29]. In the study by Green and colleagues [11], MSM hired as community liaison officers set up new social media accounts on various virtual sites such as Facebook, Badoo, gay Romeo and WhatsApp. They used these platforms to invite friends to discuss issues related to HIV prevention, use of condoms and lubricants, routine HIV testing and STI screening. They also ran several closed groups which discussed HIV, safer sex, sexuality, gender-based violence and psychosocial support needs. For MSM seeking more information or a referral, the hired MSM conducted private online and telephone conversation or accompanied the peer to the recommended service delivery clinics. According to Green and colleagues [11], the study was able to reach 15,440 MSM through social media using 3 hired MSM only. In a study by Mulongo and colleagues [29], the peer educators sent messages using coded language, through Facebook, e-mail, and SMS to encourage peers to test and ease fears about testing by counseling them. In addition, peer educators using virtual sites promoted the proper use of condoms and water-based lubricants and raised awareness on proper care of STIs.

### Community-based services delivery

Community-based service delivery was defined as the provision of health-related services to individuals or groups in the community by people who spend a substantial amount of their working time outside the health facility delivering services. In this review, we found community-based services provided by either mobile teams of healthcare providers (i.e., **mobile outreach clinics**) or by fellow MSM (peer educators) trained to provide services (i.e., **peer-led outreach**). In addition, community-based services were provided through **social events**. This review identified ten studies that reported using community-based service delivery [20–22, 24–27, 29, 31, 32].

Three studies reported using ***peer-based outreach*** in delivering services to MSM [27, 29, 30]. The interventions using this strategy involved sending peers to deliver services in settings where MSM congregated [29, 30]. MSM-peers offered HIV education, risk reduction counseling, substance use, condoms, and safer sex information. Also, they provided HIV counseling, testing, and referrals to treatment for those diagnosed HIV positive or had other sexually transmitted infections [27, 29, 30]. A study in Malawi showed that peer outreach increased MSMs’ condom use from 62.5% at baseline to 77.0% at follow-up visit 3 with a main partner (*P* = 0.02). With a casual male partner, there was an increase from 70.7% at baseline to 86.3% at follow-up 3 (*P* = 0.01). Disclosure of sexual orientation with family members increased from 25% at follow-up 1 to 55% at follow-up 3 (*p* = 0.01) [27]. A study in Nigeria compared the effect of different outreach models in increasing uptake of HIV Testing and Counseling (HTC). Results showed that MSM in the peer-led outreach arm was likely to uptake HTC compared to facility-based and integrated mobile outreach (*P* < 0.0001, AOR: 9.21; 95% CI 5.57–15.23) [30].

Of the 10 studies that employed community-based service delivery, 7 had used mobile outreach strategy [11, 22, 24–26, 29, 30]. Interventions using this strategy employed health providers in delivering services on-site (in places where MSM congregate). For example, a study in DR Congo used mobile outreach teams to provide services in the community during the day. Later, the team also delivered services at night (i.e., ***mobile “moonlight” HTC*)** as an adaptation made to increase the number of MSM and identifying those who were HIV positive [29]. Although authors did not report specific statistics on the impact of the moonlight strategy, authors explained that “moonlight mobile testing was successful in increasing testing among MSM”. Overall, 2621 MSM were reached with targeted prevention messaging in 2013 compared to 757 reached in 2012 after using both daylight and moonlight outreach service delivery. The number of MSM visiting clinics for HIV testing doubled [29].

One study provided HIV information in the township of Soweto in South Africa through conducting ***social events*** [21]. The intervention employed MSM-community leaders and trained on sexually transmitted infections, HIV as well as leadership and communication skills. The peer educators held meetings in private and safe places with MSM and discussed current events, condoms, water-based lubricants and HIV-prevention strategies. Community-based events such as sports, dance competition, and debates were used for knowledge sharing and socialization. These events enabled reaching 98 mostly gay-identified black MSM who belonged to the high-risk network [21]. The study concluded that social events and group meetings can potentially be avenues for delivering HIV information and basic supplies to MSM. Specifically, use of community-based events in Cape Town, South Africa, improved access to information, condoms, water-based lubricants and improved well-being of the of the participants [21].

### Facility-based service delivery

In this review, we define facility-based services as any health-related information, preventive or curative services delivered through an established health facility. This review identified two forms of health facilities where MSM access health services. One is the ***Local health facility***, which refers to any nearby facility providing services to the general population. The facilities may or may not be key population friendly (i.e., non-discriminatory to MSM) but provide HIV related services. The second form is the ***dedicated facility,*** which is set up by NGOs or research institutions to provide comprehensive HIV care and treatment services to MSM.

Five studies used facility-based strategy in delivering services to MSM [23, 28–30, 33]. Of the five, two [29, 30] reported linking the MSM to local facilities that were key populations-friendly (non-discriminatory to MSM). A study conducted in DR Congo reported improvement in uptake of services after starting to use a key population-friendly facility. For example, in three months (October – December 2013), the program could reach 779 MSM compared to 757 MSM reached in the whole 2012 [29]. Three studies [24, 27, 33] reported using local health centers that provided HIV preventive and treatment services as a referral facility for further health services for the MSM.

Use of **dedicated facilities** run by NGOs [26], CBOs [27, 28, 31], research institutions [20], and drop-in-centers [11, 32] proved a useful strategy in delivering services to MSM. The main reason for setting up these facilities was to provide safe and easily accessible sites with all necessary services and provide them to MSM in a non-threatening, clean and friendly environment. In some studies, peers helping this exercise received a small wage (e.g., ≈$15 each month) and telephone credit (≈$5 each month) for their work, as well as transport (≈$2) to attend clinic meetings [23, 25].

Although both public and dedicated facilities are important sources for HIV prevention and care, a study in Kenya found that MSM felt that public facilities were overly prescriptive (not patient-centered), and that providers did not understand the needs of MSM [23]. In DR Congo, the program worked to build the technical and organizational capacity of communities, health facilities and NGO to provide HIV prevention and services to key populations including MSM. Through this initiative one hospital was provided with tools and specialized equipment, including x-ray machine and proctoscopes so that MSM who test positive at HTC can be referred to the hospital for further management. With these improvements, the hospital received 779 MSM within three months (October – December 2013) in comparison to 757 MSM reached in the whole 2012 [29].

## Discussion

These findings suggest that despite a long historical record of the existence of MSM across sub-Saharan Africa [35–38], HIV interventions specifically focusing on MSM are new and limited in scope and scale. For example, 60% of identified papers reporting on HIV intervention were published in 2015 or after. Moreover, all of the studies representing East Africa (26.6%) came from Kenya with no representation from other countries. It is not surprising therefore, that reviewed studies lack clarity about how best to carry out culturally relevant HIV intervention that engages with the subjectivities that exist within this population and contexts. Also, there is limited information on strategies that can help to easily reach MSM and deliver services through existing health infrastructure and legal frameworks. These results constitute a clear call to action on intensified research and prevention interventions as proposed in other studies in a similar context [1, 39].

The interest in reporting results of interventions is to share experiences that could inform future program planning. However, results from this review show some weaknesses in reporting pertinent issues that could provide lessons to program implementers in similar contexts. For example, some studies did not show how participants were sampled [29] and engaged in the study or program [30]. Except one study [30], all studies using various service delivery strategies did not report levels of uptake for each delivery strategy separately. This limits any possibility for comparisons in terms of levels of uptake for each services delivery strategy. The lack of clear reporting of some program processes defeats the general purpose of sharing experiences and obscures the possibility for understanding the true nature of the reported results.

### A reflection on accrual strategies and lessons learned

The results of this review show that one of the strategies used to mobilize MSM is the respondent-driven sampling recruitment strategy [28, 31]. An evaluation of this approach by Baral and colleagues in recruiting MSM in Nigeria, shows that by increasing recruitment waves, the chances for mobilizing participants who have not been in contact with peer educators, nor received HIV information and services, also increases [31]. The use of this approach can be advantageous especially in contexts where MSM face legal prohibition, social stigma and discrimination. These situations force MSM to operate clandestinely [31]. Indeed, those studying hidden population such as female sex workers, MSM and illicit drug users describe respondent-driven sampling as flexible and robust method that can produce a sample representative of the heterogeneity of the target population [40, 41]. For instance, in the Nigerian study, MSM population seen in the earlier waves was different from that seen in the later waves. In the earlier waves, MSM were more likely to be educated, likely to report prior HIV testing and more likely to have initiated ART unlike those recruited in the later waves [31]. However, the effectiveness of respondent-driven sampling recruitment depends on the selection of seeds and social ties between MSM networks [42, 43].

Venue-based recruitment was another strategy used in recruiting MSM in studies included in this review. This strategy takes an advantage of the fact that some MSM tend to gather or congregate in certain locations such as bars, clubs, massage parlors and brothels [24, 29, 32]. This strategy works with venue-based peer educators in reaching and recruiting a large number of MSM at venues where it is easier to identify and access potential participants [24, 29]. However, venue-based sampling strategy has some limitations. First, due to stigma, discrimination and criminalization, the venues from which to accrue MSM participants in some settings may be very few or none. Second, the participants accrued in such venues may more likely represent individuals whose behaviors are visible, such as those who are “out” in terms of their sexual orientation and male sex workers [31, 41]. Therefore, this strategy may potentially under-represent those individuals who, due to different circumstances, are not “out” but use internet to connect with sexual partners. Third, mapping of the venues need to be comprehensive to avoid selection bias. Thus, achieving the inclusion of all sites requires sufficient time and resource commitment [44].

This analysis shows that some studies accrue potential participants by identifying them using information from the baseline studies [20, 21, 27, 32]. In this strategy, baseline study provides a sampling frame and participants are contacted and recruited by trained peers using information collected in the baseline survey. This strategy can be advantageous as it can potentially help to recruit large samples in a short amount of time and with relative minimal resources. It also makes it possible to recruit participants from specific demographics and special interest groups where necessary. In addition, the MSM accrued using this strategy may be less suspicious since they have already been exposed to the program activities. However, there are also limitations to recruitment using information from baseline or pre-existing studies. The use of participant information from baseline studies relies heavily on the credibility and rigor of the baseline data collection process. This could potentially be an issue where participant tracer or locator information was not thoroughly obtained in the baseline study. Moreover, considering the continuing stigma and laws that criminalize MSM, most tend to be mobile [29], which may limit assurance for reaching them.

Engagement of grassroots MSM organizations provides an important pool of MSM for studies and interventions [22, 25, 26]. The advantage of working with NGOs is that they are trusted and provide assurance for the safe and stigma-free environment. This is consistent with findings from other studies which show that involvement in NGO activities can reduce their isolation, enhance their self-esteem, and improve community perceptions about this population [45, 46]. Nevertheless, use of NGOs as the only source of participants for intervention has the risk of missing out potential beneficiaries of the intervention or research because they may be out of the circles of those that are served by the NGO. Furthermore, there is a possibility that those associated with NGOs are those who are “out” and identify themselves as homosexuals something which under-represents the MSM who are not “out”. Moreover, funding for NGOs may be short-term, thus this may not be a sustainable option for long-term interventions. It may therefore be important to expand accrual of MSM for HIV interventions and research beyond those whom grassroots organizations serve to increase program reach and impact.

Use of virtual sites has shown to be a useful strategy in reaching MSM [11, 29, 33] if a complete list of virtual sites is identified by MSM peer leaders. Results in this review show that use of virtual sites helped programs to reach many MSM compared to traditional strategies. For instance, a Ghanaian study reached 15,440 unique MSM through social media and 12,804 MSM through peer outreach activities in the same period of implementation [11]. The benefit of using virtual sites for recruitment of MSM is that it greatly expands the reach to those who may not be reached by traditional means such as venue-based or peer-based strategies because of stigma and criminalization [11, 29]. For instance, only 18% and 27% of the MSM reached via online platforms in Accra and Kumasi respectively were also reached by peer educators in the same period [11]. The social aspect inherent to virtual sites allows for enrolled participants to recruit others for research, thus minimizing the time and monetary expenses which would otherwise be required to reach large samples. The challenge with this strategy is that it may not be used in the rural settings where use of online platforms is limited. For example, although all included studies that used virtual site strategy in reaching MSM reported success, all of them were conducted in cities and urban settings where there are platforms for the virtual domain [11, 29, 33]. There is a dearth of evidence on whether virtual site strategy for reaching MSM can equally be feasible in rural settings of sub-Saharan Africa.

### A reflection on delivery and lessons learned

Peer-led outreach plays a critical role in generating demand in the community by tapping into MSM social networks. This analysis shows that use of peer-led outreach enables MSM to access services confidently and remaining into care [27]. The main reason is that MSM trust their peers, and become comfortable with service providers associated or known by a peer educator [12]. Studies in other settings have shown that use of peer educators improves HIV knowledge, access, and uptake of HIV preventive and treatment services among MSM [47–49]. These findings, therefore, suggest that health promotion activities that use peer educators are likely to be successful in sub-Saharan Africa because of the contextual challenges MSM face in their day-to-day lives.

In sub-Saharan Africa, use of available health facilities would be helpful in reaching as many MSM as possible with HIV information and services because of their coverage. However, results of this review show that facilities need to be equipped with provider skills and equipment. Stigma reduction programs and skills in providing MSM-friendly services among health providers are necessary to increase trust and acceptability to MSM. This review shows that improved facilities better support individuals who test HIV positive move through the HIV treatment cascade and safely receive treatment without discrimination [29]. Indeed, studies that have tried to influence institutional level factors have provided evidence of improvement in service delivery and uptake of services [10]. Thus, apart from behavioral and biomedical interventions provided, enabling facilities to provide services may help to transcend challenges related to sustainability and improved linkage to care.

In this review, even though moonlight service delivery has shown potential to increase the program reach and uptake of services, providing follow-up services may be a challenge because of mobility of MSM [29]. In DR Congo, use of this method failed to offer follow-up services to both HIV positive and negative MSM. In addition, stigma and criminalization of same-sex relationships make security for service providers and participants a major concern.

This review shows that virtual sites can be a potential platform for reaching MSM across SSA. However, delivery of HIV services through virtual methods can be limited to information or education as other services such as HIV testing and treatment, require direct contact with the service provider. Studies conducted on virtual methods have shown that although this strategy targets a population of interest, there is usually a steep drop-out of participants if they are asked to engage in intensive programs [50, 51]. Experience elsewhere shows that about half of participants recruited for an HIV prevention study from Facebook dropped out of study after being invited for the full study post-screening [52]. These experiences should raise awareness among programmers considering HIV intervention among MSM in SSA where stigma and criminalization is high.

### Strengths and limitations

The strength of this review is its primary focus on accrual of MSM to take part in HIV prevention interventions and HIV service delivery strategies. Two authors screened studies for eligibility, and another author helped at times of difference, which assured accuracy, reliability, and transparency. Nevertheless, this review has some limitations. First, as this review is set out to summarize accrual and delivery strategies for MSM in SSA, neither does it attempt to show the effectiveness of these approaches with respect to the delivery of interventions nor specific accrual strategies relate to differential service delivery success. Second, this review shows only absolute numbers of participants reached by different strategies as reported in the reviewed studies and does not try to suggest the suitable approaches as differentiated by MSMs’ characteristics. Third, because this review obtained studies published in the English language only, there may be possibilities for missing out other relevant studies and insights from the Francophone Africa and Portuguese speaking countries. Also, the search for relevant studies was limited to peer-reviewed journals only, which may miss relevant program reports, conference presentations, and book chapters. The diversity of studies included in this review by design and methods can also influence interpreting the results. Conducting a meta-analysis or a meta-ethnographic synthesis for this review was not possible as studies included were different in designs and methods.

## Conclusions

As calls for increasing HIV prevention efforts among key populations including MSM are mounting, a synthesis of MSM accrual methods and service delivery strategies is crucial due to stigma and laws criminalizing same-sex practices and relationships in most of sub-Saharan Africa. Our review shows that MSM can be accrued to HIV prevention interventions using strategies such as respondent driven sampling, venue-based approaches, engagement with existing or previous programs or studies and virtual based platforms. All accrue methods reviewed relied on the existence of the prior-contact between peers or NGO workers and individuals reporting same-sex relationships. The trusting relationship between MSM and people recruiting them for HIV interventions create an enabling environment for effective accrual.

The HIV prevention interventions can be delivered to MSM using strategies including facility-based, community-based as well as virtual strategies. Most intervention reviewed used dedicated or MSM friendly facilities highlighting stigmatizing setting in which MSM operate. Some studies accrued and tested for HIV infection in the community but referred MSM to health facilities for further HIV services. Taken together, the evidence suggests that there are various strategies for accrual and delivering services to MSM across SSA. However, each of these strategies have specific strengths and weaknesses necessitating combinations of interventions and integration of the specific context to inform implementation. If the best of intervention content and implementation are used to inform these services, sufficient coverage and impact of HIV prevention and treatment programs for MSM across Sub Saharan Africa can be optimized.

## Additional file

Additional file 1: A combined search strategy in MEDLINE. This file presents an illustrative search strategy as conducted on MEDLINE database. (DOC 30 kb)

## Abbreviations

MSM: Men who have sex with men
RDS: Respondent-driven Sampling
SSA: sub-Saharan Africa
STIs: Sexually transmitted infections
