# Supplementary material for: Participants’ accrual and delivery of HIV prevention interventions among men who have sex with men in sub-Saharan Africa: a systematic review
Source: BMC Public Health. 2018 Mar 20;18:370. doi: 10.1186/s12889-018-5303-2 (PMC5859521; doi:10.1186/s12889-018-5303-2)
Supplement: Supplementary file 1 — A combined search strategy in MEDLINE. This file presents an illustrative search strategy as conducted on MEDLINE database. (DOC 30 kb) [file 12889_2018_5303_MOESM1_ESM.doc]

Additional file 1: A combined search strategy in MEDLINE

| TOPIC ("hiv"[MeSH Terms] OR "hiv"[All Fields]) OR "HIV infection"[All Fields] OR ("hiv-1"[MeSH Terms] OR "hiv-1"[All Fields] OR "hiv 1"[All Fields]) OR ("hiv-2"[MeSH Terms] OR "hiv-2"[All Fields] OR "hiv 2"[All Fields]) OR ("hiv"[MeSH Terms] OR "hiv"[All Fields] OR ("human"[All Fields] AND "immunodeficiency"[All Fields] AND "virus"[All Fields]) OR "human immunodeficiency virus"[All Fields]) OR ("acquired immunodeficiency syndrome"[MeSH Terms] OR ("acquired"[All Fields] AND "immunodeficiency"[All Fields] AND "syndrome"[All Fields]) OR "acquired immunodeficiency syndrome"[All Fields]) OR (("hiv"[MeSH Terms] OR "hiv"[All Fields]) AND type[All Fields] AND 1[All Fields]) OR ("hiv-2"[MeSH Terms] OR "hiv-2"[All Fields] OR "hiv type 2"[All Fields])  **POPULATION**  "men who have sex with men"[All Fields] OR "MSM"[All Fields] OR ("male"[MeSH Terms] OR "male"[All Fields]) AND who[All Fields] AND ("sex"[MeSH Terms] OR "sex"[All Fields]) AND ("male"[MeSH Terms] OR "male"[All Fields]) OR ("bisexuality"[MeSH Terms] OR "bisexuality"[All Fields] OR "bisexual"[All Fields]) AND ("men"[MeSH Terms] OR "men"[All Fields]) OR "homosexuality, male"[MeSH Terms] OR ("homosexuality"[All Fields] AND "male"[All Fields]) OR "male homosexuality"[All Fields] OR ("male"[All Fields] AND "homosexual"[All Fields]) OR "male homosexual"[All Fields] OR gay[All Fields] AND ("men"[MeSH Terms] OR "men"[All Fields] OR "man"[All Fields])  **CONTEXT**  "Africa south of the Sahara"[MeSH Terms] OR ("Africa"[All Fields] AND "south"[All Fields] AND "Sahara"[All Fields]) OR "Africa south of the Sahara"[All Fields] OR ("sub"[All Fields] AND "Saharan"[All Fields] AND "Africa"[All Fields]) OR "sub Saharan Africa"[All Fields]  **PERIOD**  ("2007/03/01"[PDAT] : "2017/04/01"[PDAT]) |
| --- |
